# Relative Effect Potency Estimates of Dioxin-like Activity for Dioxins, Furans, and Dioxin-like PCBs in Adults Based on Two Thyroid Outcomes

**DOI:** 10.1289/ehp.1205739

**Published:** 2013-05-10

**Authors:** Tomáš Trnovec, Todd A. Jusko, Eva Šovˇcíková, Kinga Lancz, Jana Chovancová, Henrieta Patayová, L’ubica Palkoviˇcová, Beata Drobná, Pavel Langer, Martin Van den Berg, Ladislav Dedik, Soˇna Wimmerová

**Affiliations:** 1 Slovak Medical University, Bratislava, Slovakia; 2 Epidemiology Branch, National Institute of Environmental Health Sciences, National Institutes of Health, Department of Health and Human Services, Research Triangle Park, North Carolina, USA; 3 Institute of Experimental Endocrinology, Slovak Academy of Sciences, Bratislava, Slovakia; 4 Institute for Risk Assessment Sciences, Utrecht University, Utrecht, the Netherlands; 5 Faculty of Mechanical Engineering, Slovak University of Technology in Bratislava, Bratislava, Slovakia

**Keywords:** dioxin-like polychlorinated biphenyl (DL-PCB), free thyroxine (FT_4_), polychlorinated dibenzo-*p*-dioxins (PCDDs), polychlorinated dibenzo-*p*-furans (PCDFs), relative effect potency (REP), thyroid volume, toxic equivalency factor (TEF)

## Abstract

Background: Toxic equivalency factors (TEFs) are an important component in the risk assessment of dioxin-like human exposures. At present, this concept is based mainly on *in vivo* animal experiments using oral dosage. Consequently, the current human TEFs derived from mammalian experiments are applicable only for exposure situations in which oral ingestion occurs. Nevertheless, these “intake” TEFs are commonly—but incorrectly—used by regulatory authorities to calculate “systemic” toxic equivalents (TEQs) based on human blood and tissue concentrations, which are used as biomarkers for either exposure or effect.

Objectives: We sought to determine relative effect potencies (REPs) for systemic human concentrations of dioxin-like mixture components using thyroid volume or serum free thyroxine (FT_4_) concentration as the outcomes of interest.

Methods: We used a benchmark concentration and a regression-based approach to compare the strength of association between each dioxin-like compound and the thyroid end points in 320 adults residing in an organochlorine-polluted area of eastern Slovakia.

Results: REPs calculated from thyroid volume and FT_4_ were similar. The regression coefficient (β)-derived REP data from thyroid volume and FT_4_ level were correlated with the World Health Organization (WHO) TEF values (Spearman *r* = 0.69, *p* = 0.01 and *r* = 0.62, *p* = 0.03, respectively). The calculated REPs were mostly within the minimum and maximum values for *in vivo* REPs derived by other investigators.

Conclusions: Our REPs calculated from thyroid end points realistically reflect human exposure scenarios because they are based on chronic, low-dose human exposures and on biomarkers reflecting body burden. Compared with previous results, our REPs suggest higher sensitivity to the effects of dioxin-like compounds.

## Introduction

Polychlorinated dibenzo-*p*-dioxins (PCDDs, dioxins), polychlorinated dibenzofurans (PCDFs, furans), and polychlorinated biphenyls (PCBs) are ubiquitous environmental compounds. PCDDs and PCDFs are combustion or industrial by-products with no commercial use, whereas PCBs have been frequently used in a variety of commercial applications, such as coolants and lubricants in transformers, capacitors, and other electrical equipment. Some PCBs act in a manner mechanistically similar to that of 2,3,7,8-tetrachlorodibenzo-*p*-dioxin (TCDD); these PCBs are usually referred to as dioxin-like PCBs (DL-PCBs). PCDDs, PCDFs, and PCBs are commonly found in mixtures in the environment and human food chain, usually containing a large number of congeners, such that each mixture has its own degree of dioxin-like toxicity. For risk assessment purposes, the World Health Organization (WHO) assigned each of these individual compounds a toxic equivalency factor (TEF) value relative to the toxicity of TCDD (Van den Berg et al. 2006). This factor indicates a relative toxicity compared to the most toxic congener, TCDD, which is given a reference value of 1. Prerequisites for this TEF concept are the exclusive inclusion of toxic effects that are mediated via the aryl hydrocarbon receptor (AhR) and an additive mechanism of action for mixtures of these compounds. Otherwise, mediated toxic effects of PCDDs, PCDFs, and PCBs cannot be quantified for risk assessment by this method.

The reevaluation of TEF values for these compounds has become a continuous process based on available results from *in vivo* and *in vitro* studies. Although many studies using human cell lines or primary cells have been published to date (Haws et al. 2006), human *in vivo* data that may contribute to the TEF concept have not been published previously. In an attempt to fill this gap, we examined cross-sectional data on thyroid impairment in a population exposed to a mixture of organochlorines to identify relationships between individual mixture components and thyroid volume and free thyroxine (FT_4_). Based on these results, we estimated the relative potencies (REPs) of PCDD, PCDF, and DL-PCB congeners in adult humans.

## Materials and Methods

*Participants*. Our initial sample of 2,047 adults was drawn from a population living in the towns and villages of the Michalovce, Svidnik, and Stropkov districts in eastern Slovakia, an area known to be contaminated by a mixture of organochlorines (Jursa et al. 2006; Langer et al. 2007c; Petrik et al. 2006). Adult participants were recruited between August 2001 and February 2002 with the help of primary care physicians, who randomly selected names from alphabetical lists of their patients; nearly all those approached agreed to participate. We have complied with all applicable requirements of U.S. and international and national regulations. The study protocol was approved by the institutional review board of the Slovak Medical University. All human participants gave written informed consent prior to the study.

Although we did not collect data on place of birth, we assumed that all participants spent most of their adult life residing in these districts, which is in agreement with low labor mobility in Slovakia. Individuals having a mild, chronic controlled illness (e.g., rheumatic diseases, hypertension, diabetes, thyroid disorders, non-morbid obesity, allergy) were not excluded from the study. At enrollment, participants were given a physical examination by our field medical staff, and sociodemographic and medical questionnaires were completed (Langer 2010; Langer et al. 2006, 2007a, 2007b, 2007c, 2008; Rádiková et al. 2008; Ukropec et al. 2010).

Whole blood samples were collected from fasting participants into anticoagulant-free Vacutainer™ tubes (S-Monovette; Sarstedt, Nürnberg, Germany); after clotting, samples were centrifuged at 3,000 rpm for 15 min. The serum was frozen in glass vials and stored at –18°C.

*Chemical analyses.* Of the 2,047 adults selected, 320 were willing to provide 90 mL of blood for analysis of PCDDs, PCDFs, and PCBs. The serum samples were treated using a modified version of the method by Turner et al. (1994). Each thawed serum sample (5–30 mL) was spiked with ^13^C_12_-labeled standards [15 2,3,7,8-substituted PCDDs/PCDFs, 12 DL-PCBs, and 11 non-dioxin-like (NDL)-PCBs (Cambridge Isotope Laboratories Inc., Andover, MA, USA; Wellington Laboratories Inc., Ontario, Canada)] 24 hr before sample processing. After the serum was treated with diluted formic acid, the analytes were isolated by solid phase extraction using a 10-g C18 column (UCT Inc., Bristol, PA, USA). A hexane extract was cleaned on a Power-PREP™ semiautomated clean-up system (FMS Inc., Waltham, MA, USA) with prepacked disposable silica, alumina, and carbon columns. A combined dichloromethane/*n*-hexane (2:98, vol/vol) and dichloromethane/*n*-hexane (50:50, vol/vol) eluate fraction contained mono-*ortho* and NDL-PCBs. A toluene eluate fraction contained PCDDs/PCDFs and non-*ortho* PCBs. The eluate fractions were concentrated and then diluted with ^13^C_12_-labeled recovery standards.

We used an HP 6890 Plus gas chromatograph (Hewlett-Packard, Palo Alto, CA, USA) coupled with an MAT 95XL mass spectrometer (Thermo Finnigan, Bremen, Germany) operating at a 10% valley resolution of 10,000 in the selected ion monitoring mode to identify and measure 2,3,7,8-substituted PCDDs/PCDFs and PCBs. PCDD/PCDF and non-*ortho*-PCB congeners were separated on a 30 m × 0.25 mm × 0.25 µm DB-5ms capillary column (J&W Scientific, Folsom, CA, USA), and mono-*ortho* and NDL-PCB congeners were separated on a 60 m × 0.25 mm × 0.25 µm DB-5ms capillary column (J&W Scientific). The qualitative and quantitative analyses were carried out using U.S. Environmental Protection Agency (EPA) isotope dilution methods 1613 (U.S. EPA 1994) and 1668 (U.S. EPA 1999). Two congeners [1,2,3,7,8,9-hexachlorinated CDF (HxCDF) and PCB 77] were not included in the statistical analysis: 1,2,3,7,8,9-HxCDF because concentrations were below the limit of detection (LOD; 0.22–3.1 pg/g lipid) in all analyzed samples, and PCB 77 because of high background levels in laboratory blanks.

All analytical measurements were carried out at the National Reference Centre for Dioxins and Related Compounds (Department of Toxic Organic Pollutants, Slovak Medical University), which has been certified by the Slovak National Accreditation Service (ISO/IEC 17 025:2005, certification No. S-111) and regularly participates in interlaboratory studies and proficiency tests on dioxins and PCBs in food and feed. Each analysis batch consisted of 14 serum samples, 1 method blank, and 1 quality control (QC) sample (porcine serum spiked with native PCDD, PCDF, and PCB congeners as an in-house reference material). Certified human serum [SRM (standard reference material) 1589a; National Institute of Standards and Technology, Gaithersburg, MD, USA] was analyzed in each third batch (Kočan et al. 2004). Control charts were plotted for QC samples, blanks, and verification calibration standards to check accuracy, precision, and reliability of the analytical process.

We used an enzymatic method based on the determination of total cholesterol, free cholesterol, phospholipids, and triglycerides (Akins et al. 1989) to determine total lipids in all of the serum samples analyzed. We used these values to present the organochlorine concentrations on a lipid weight basis.

*Assessment of thyroid outcomes.* Thyroids were examined and measured using a portable Sonoline SI-400 diagnostic ultrasound device (Siemens, North Rhine-Westphalia, Germany) with a 7.5-MHz linear transducer. For thyroid measurement, each participant lay supine with the neck hyperextended. The thyroid volume (in milliliters) for each lobe was calculated according to the following ellipsoid formula: width (in centimeters) × length (in centimeters) × thickness (in centimeters) × a correction factor of 0.479 (Brunn et al. 1981). All measurements were performed by the same physician who had long-term experience in field surveys and clinical ultrasound diagnostics. The physician was unaware of toxicant concentrations among participants. We estimated the intraobserver variation as described by (Ozgen et al. 1999) using three separate measurements of 50 thyroid volumes (representing 50 participants) that ranged from 3.0 to 20.5 mL; the mean ± SD was 3.9 ± 3.5%, and the median was 6.2 mL.

FT_4_ was determined in stored serum specimens using an automated electrochemiluminescent immunoassay system (Elecsys system; Roche, Basel, Switzerland), as described previously (Langer et al. 2005).

*Statistical analysis.* We used two approaches to estimate the relative potencies of individual components of the mixture. The first approach, as suggested previously by Fattore et al. (2004) and Yang et al. (2010), was based on a comparison of benchmark concentrations (BMCs) calculated for thyroid outcomes as a function of organochlorine serum concentrations. The second approach was based on comparing the magnitude of the regression coefficient (β) for thyroid volume or FT_4_ serum concentration regressed on the serum concentration of the individual congeners, similar to the study by Brown et al. (2001). Participant’s sex and age at blood draw, as well as PCDDs, PCDFs, and PCBs determined in the exposure mixture were potential confounders. Thus, in multivariable regression to calculate REPs, we adjusted for sex, age, and presence of other organochlorines. For confirmation of TCDD as an index (reference) congener (U.S. EPA 2000), we used multiple regression with backward elimination (variable removal at *p* > 0.1). We compared the REPs resulting from both approaches with published data on REPs for DLCs (Haws et al. 2006) and with WHO-TEF values (Van den Berg et al. 2006).

*Estimation of REPs through comparison of BMCs.* For each individual PCDD, PCDF, and DL-PCB congener, we calculated the BMC (Crump 1995) for thyroid volume and FT_4_ serum concentration end points, using CTDB_BMD software (Dedik 2012). We adjusted for sex and age in all statistical models. The BMCs for changes in thyroid volume and serum FT_4_ associated with TCDD concentration were compared with the BMCs of individual congeners and used to derive the congener-specific REPs. Thus, BMC_TCDD_/BMC*_i_* is the relative potency (REP*_i_*) for the *i*th congener, relative to TCDD.

*Estimation of REPs through comparison of regression coefficients.* We calculated the regression coefficient (β) for each congener from all concentration data > LOD. We considered sex and age, along with PCDD, PCDF, and PCB congeners identified in the mixture, as confounding variables. We calculated the BMCs for the most probable combinations of confounders [see Supplemental Material, Table S1 (http://dx.doi.org/10.1289/ehp.1205739) for a list of those with the greatest influence on BMCs]. However, because the addition of other organochlorines had negligible influence on model data, we present results with adjustment only for age and sex. The REPs of the individual congeners were calculated as the ratio of β coefficient obtained for the *i*th congener to β⊇coefficient for TCDD: β*_i_*/β_TCDD_.

## Results

*Participant characteristics*. The subgroup of 320 participants with complete data consisted of 203 males 44.9 ± 11.47 years of age (mean ± SD; median, 48 years) and 127 females 47.3 ± 9.24 years of age (median 48 years), with an overall mean age of 45.8 ± 10.7 years (median, 48 years). Among males, the age range was 20–75 years, and in females 21–70 years. The median and mean serum concentrations (in picograms WHO TEQ per gram lipid) of DLCs in these participants are shown in Supplemental Material, Table S2 (http://dx.doi.org/10.1289/ehp.1205739). Data on mean and median serum concentrations of PCDDs, PCDFs, DL-PCB congeners, and the most abundant NDL-PCB congeners from samples with concentrations > LOD are shown in Supplemental Material, Table S3. The median concentrations of individual congeners with concentrations > LOD correlated with median concentrations overlapping with TCDD > LOD (*r* = 0.998). Thus, we assumed that parameters calculated from samples overlapping with TCDD > LOD well represent those from samples > LOD.

For males and females, the mean (± SD) of volume of the thyroid gland were 11.56 ± 4.42 mL (median, 10.20) and 9.49 ± 4.75 mL (median, 8.35), respectively. Mean (± SD) serum concentrations of FT_4_ for males and females were 16.93 ± 2.65 pmol/L (median, 16.7) and 15.72 ± 3.22 pmol/L (median, 15.39), respectively.

*Identification of the index congener.* There is general agreement that an index compound should be the most well-studied member of its class and that it should provide the largest body of acceptable scientific data (U.S. EPA 2000). At the same time, an index chemical should be potent with regard to the expected end point. We used multiple regression with backward elimination to query the selection of TCDD as the index congener in concurrence with other PCDD or PCDF congeners. We created four models (A–D) for this purpose. When we entered thyroid volume as the dependent variable and concentrations of the seven most toxic PCDD congeners [TCDD, 1,2,3,7,8-pentachlorinated CDD (PeCDD), 1,2,3,4,7,8-HxCDD, 1,2,3,6,7,8-HxCDD, 1,2,3,7,8,9-HxCDD, 1,2,3,4,6,7,8-heptachlorinated CDD (HpCDD), and octachlorinated CDD (OCDD)] as independent variables, cross-tabulation for samples > LOD reduced the number of individuals to an insufficient 25. If we omitted the two HxCDD congeners with relatively low concentrations (1,2,3,4,7,8-HxCDD and 1,2,3,7,8,9-HxCDD), the study population increased to 62 individuals. Model A showed that with respect to thyroid volume reduction, TCDD was the most potent congener [see Supplemental Material, Table S4 (http://dx.doi.org/10.1289/ehp.1205739)]. In model D, with FT_4_ as the end point of interest, multiple regression eliminated four PCDF congeners when they were combined with TCDD (see Supplemental Material, Table S4). However, multiple regression did not confirm the role of TCDD with FT_4_ as the dependent variable and PCDD congeners as the independent variable (see Model B in Supplemental Material, Table S4) or with thyroid volume as the dependent variable and PCDF congeners as the independent variable (see Model C in Supplemental Material, Table S4).

*Assessment of REPs for PCDDs, PCDFs, and DL-PCBs.* Data in Table 1 show that PCDDs were associated with a decrease in both thyroid volume and FT_4_ level. The association between thyroid volume and dioxins decreased with the increasing number of chlorine substitutes in the compound, except for 1,2,3,7,8,9-HxCDD. The PCDFs were associated with a decrease in thyroid volume in a similar manner except for two compounds (1,2,3,4,7,8-HxCDF and OCDF). With respect to FT_4_, we observed a mixed response: There was a negative association with 2,3,7,8-tetraCDF (TCDF), 2,3,4,7,8-PeCDF, 1,2,3,4,6,7,8-HpCDF, and OCDF and a positive association with 1,2,3,7,8-PeCDF and the three HxDF congeners (1,2,3,4,7,8-HxCDF, 1,2,3,6,7,8-HxCDF, and 2,3,4,6,7,8-HxCDF). The DL-PCBs were related to an increase in both thyroid volume and FT_4_ serum level, except for the non-*ortho*-substituted congener PCB 81 for both thyroid volume and FT_4_ and the mono-*ortho*-substituted congener PCB 105 for FT_4_. Of all the congeners, TCDD was most strongly associated with a decrease of thyroid volume and FT_4_ level. NDL-PCBs were associated with slight changes, compared with TCDD, appearing as increases with the most abundant PCB congeners [see Supplemental Material, Table S5 (http://dx.doi.org/10.1289/ehp.1205739)]. To comply with the assumption that congeners have a similar mode of action (U.S. EPA 2000), we calculated the REPs only for those acting in the same direction as the index chemical. Thus, congeners associated with an increase of thyroid volume or FT_4_ level were not further analyzed.

**Table 1:** The calculated REPs of PCDD, PCDF, and DL-PCB congeners.

Congener	*n*	Thyroid volume				FT_4_				WHO TEF^*e *^	REP_2004_ database^*a *^		
		β	REP^*b *^as β_*i*_/β_TCDD_	REP^*c *^as BMCL	REP^*d*^ as BMC	β	REP^*b *^as β_*i*_/β_TCDD_	REP^*c *^as BMCL	REP^*d*^**as BMC		Minimum	Median	Maximum
PCDDs
2,3,7,8-TCDD	70	–1.101	1	1	1	–0.508	1	1	1	1			
1,2,3,7,8-PeCDD	132	–0.45	0.432	0.143	0.325	–0.24	0.471	0.907	0.847	1	0.044	0.4	1.5
1,2,3,4,7,8-HxCDD	81	–0.283	0.257	0.237	0.332	–0.409	0.805	1.413	0.981	0.1	0.0076	0.059	0.35
1,2,3,6,7,8-HxCDD	286	–0.091	0.082	0.049	0.085	–0.064	0.126	0.238	0.256	0.1	—	—	—
1,2,3,7,8,9-HxCDD	76	0.146				–0.245	0.482	1.603	0.853	0.1	0.029	0.029	0.029
1,2,3,4,6,7,8-HpCDD	316	–0.009	0.008	0.014	0.011	–0.015	0.029	0.065	0.068	0.01	0.001	0.01	0.035
OCDD	319	–0.003	0.003	0.002	0.001	0.002				0.0003	0.00025	0.00025	0.00025
PCDFs
2,3,7,8-TCDF	43	–0.912	0.828	0.629	0.635	–0.051	0.1	0.685	0.128	0.1	—	—	—
1,2,3,7,8-PeCDF	13	–0.382	0.347			0.657				0.03	0.0027	0.022	0.95
2,3,4,7,8-PeCDF	314	–0.019	0.016	0.011	0.016	–0.01	0.02	0.02	0.03	0.3	0.0065	0.2	3.7
1,2,3,4,7,8-HxCDF	311	0.023				0.043				0.1	0.014	0.05	0.16
1,2,3,6,7,8-HxCDF	312	–0.161	0.146	0.067	0.091	0.012				0.1	0.0031	0.081	0.16
2,3,4,6,7,8-HxCDF	51	–0.86	0.78	0.257	0.322	1.084				0.1	0.015	0.018	0.1
1,2,3,4,6,7,8-HpCDF	314	–0.059	0.054	0.083	0.132	–0.027	0.053	0.194	0.083	0.01	—	—	—
OCDF	80	0.127				–0.19	0.373	0.367	0.136	0.0003	0.000004	0.000077	0.0016
DL-PCBs
PCB 81	234	–0.0111	0.01	0.011	0.025	–0.009	0.017	0.041	0.05	0.0003	—	—	—
PCB 126	319	0.0009				0.000040				0.1	0.000067	0.1	0.86
PCB 169	320	0.0034				0.0022				0.03	0.0000018	0.019	0.74
PCB 105^*f*^	276	0.0096				–0.0009	0.0000019^*g*^	0.000024^*g*^	0.000007^*g*^	0.00003	0.00000047	0.000042	0.0022
PCB 114^*f*^	315	0.063				0.0213				0.00003	0.0002	0.00034	0.00048
PCB 118^*f*^	301	0.0032				0.0005				0.00003	0.00000042	0.00002	0.0023
PCB 123^*f*^	276	0.033				0.022				0.00003	0.000034	0.000044	0.000055
PCB 156^*f*^	315	0.0075				0.0022				0.00003	0.0000021	0.000055	0.42
PCB 157^*f*^	315	0.0291				0.0087				0.00003	0.000420	0.0011	0.0017
PCB 167^*f*^	315	0.0192				0.0027				0.00003	—	—	—
PCB 189^*f*^	315	0.0265				0.0117				0.00003	0.000037	0.000055	0.00018
Abbreviations: BMC, benchmark concentration; BMCL, benchmark concentration lower confidence limit; DL-PCB, dioxin-like PCB; REP, relative effect potency. β, BMCL, and BMC values were adjusted for sex and age. All REPs were calculated from data in picograms per gram. ^***a***^Data from Haws etal. (2006). ^***b***^Calculated as the ratio of the β of the individual chemical to that of TCDD. ^***c***^Calculated as the ratio of the BMCL of the individual chemical to that of TCDD. ^***d***^Calculated as the ratio of the BMC of the individual chemical to that of TCDD. ^***e***^Data from VandenBerg etal. (2006). ^***f***^All β values were calculated from data in picograms per gram except for mono-*ortho*-substituted PCBs, which were in nanograms per gram. ^***g***^Calculated from data in picograms per gram.													

Sex and age were included as confounders (confounders 1 and 2) in all analyses. To assess the effect of confounding by other DLC congeners identified in the exposure mixture on β coefficients, we computed BMCs for thyroid volume decrease related to the serum concentration of individual congeners and entered the various combinations of congener confounders. We set both *p*_0_ (the background risk at zero concentration) and benchmark response (BMR) at 0.1, which translates to an increase in risk of 200% (Crump 1995). Based on the Akaike information criterion, we used these two regression models: *f*(*t*) = *a*_1_ + *a*_2_*t*, and *f*(*t*) = *a*_1_ + *a*_2_*t*^2^.

We observed that the BMC and the BMC lower confidence limit (BMCL) for TCDD were slightly influenced by the presence of other congeners in the exposure mixture [see Supplemental Material, Table S1, confounders 3–6 (http://dx.doi.org/10.1289/ehp.1205739)]. In addition, when TCDD was entered as a confounder in combination with other congeners (e.g., with the second most potent congener, 1,2,3,7,8-PeCDD), we obtained similar results. Neither of these adjustments for PCB congeners affected the BMC and BMCL value of TCDD. Therefore, in Table 1, we present REPs that were derived after adjusting only for sex and age.

The REPs in Table 1 were calculated as the relation of the individual congener β*_i_*, BMC*_i_*, or BMCL*_i_* of to the β_TCDD_, BMC_TCDD_, or BMCL_TCDD_, respectively, of the index chemical. The REPs calculated using β coefficient, BMC, and BMCL data correlated strongly between themselves (all *r*-values were > 0.903, *p* < 0.0001). Moreover, we observed a strong correlation between the REPs calculated from the largely independent thyroid volume and FT_4_ data. The Spearman correlations (*r*_S_) for REPs were derived from thyroid volume and FT_4_ data using the β_i_/β_TCDD_ (*r*_S_ = 0.81, *p* = 0.015), BMC (*r*_S_ = 0.786, *p* = 0.021), and BMCL (*r*_S_ = 0.857, *p* = 0.007) approaches.

**Figure 1:**
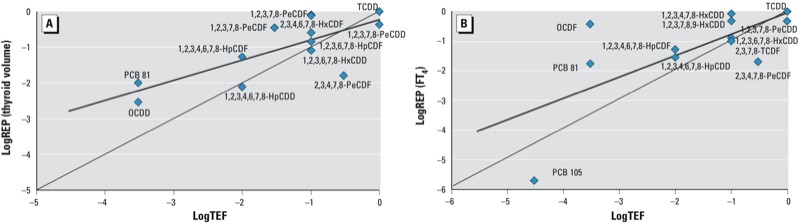
Relationship between β coefficient–derived REPs for individual mixture components and the published WHO TEFs (Van den Berg et al. 2006), as measured by (*A*) thyroid volume and (*B*) serum FT_4_.

As shown in Figure 1, the β coefficient–derived REP data for thyroid volume and FT_4_ level (β*_i_*/β_TCDD_ column in Table 1) correlated significantly with the WHO TEF values (Van den Berg et al. 2006) (thyroid volume, *r*_S_ = 0.693, *p* = 0.009; FT_4_, *r*_S_ = 0.616, *p* = 0.033), The best fit is logREP = 0.566, logTEF = –0.229 for thyroid volume and logREP = 0.363, logTEF = –0.399 FT_4_. According to our estimates, the potencies of congeners above the central axis are greater than the TEFs, and vice versa. The BMC- and BMCL-derived REP data correlated less significantly with the WHO TEF values (data not shown).

To show our REPs in a broader context, we included in Table 1 the minimum, maximum, and median values published for *in vivo* REPs in the REP_2004_ database (see Table 8 of Haws et al. 2006). Our REPs for all PCDD congeners studied and thyroid volume outcome [note that data on 1,2,3,6,7,8-HxCDD were not included by Haws et al. (2006)], irrespective of the method of derivation, are between the maximum and minimum values estimated by other researchers, except for those of OCDD. Our REPs for 1,2,3,4,7,8-HxCDD and 1,2,3,7,8,9-HxCDD, where FT_4_ is the outcome, were higher than the published maximum estimates (Haws et al. 2006). Of the three REP values (β*_i_*/β_TCDD_, BMCL, and BMC) for 1,2,3,4,6,7,8-HpCDD, the β*_i_*/β_TCDD_ ratio (0.029) is smaller than the published maximum estimate (0.035) (Haws et al. 2006). For PCDF congeners associated with thyroid volume, the REPs were close to the maximum values determined by other investigators, except for 2,3,4,7,8-PeCDF, which is higher than the minimum reported value of 0.0065 (Haws et al. 2006). We calculated REPs for four PCDF congeners with FT_4_ as an outcome; for two of them (2,3,7,8-TCDF and 1,2,3,4,6,7,8-HpCDF), values were unavailable for comparison. However, the REP for 2,3,4,7,8-PeCDF fits within the range published by Haws et al. (2006), whereas the REP for OCDF is an outlier with regard to TEFs.

**Figure 2:**
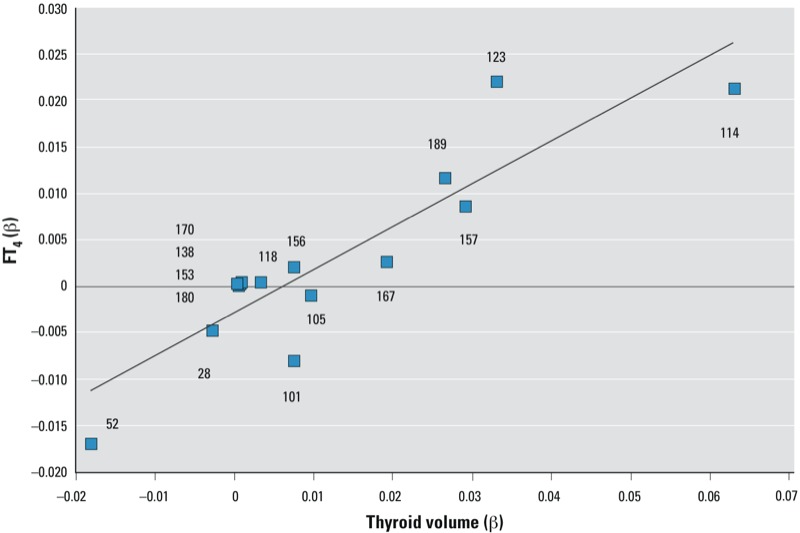
Plot of regression coefficients [β; listed in Table 1; see also Supplemental Material, Table S5 (http://dx.doi.org/10.1289/ehp.1205739)] for thyroid volume vs. PCB congener concentration (x‑axis) against those for FT_4_ serum concentration vs. PCB congener concentration (y‑axis).

When analyzing the relative magnitude of thyroid effects of PCB congeners, we included both DL-PCB and NDL-PCB congeners. Figure 2 shows plotted β values for PCB congeners for thyroid volume against those for FT_4_ serum level shown in Table 1 [see also Supplemental Material, Table S5 (http://dx.doi.org/10.1289/ehp.1205739)]. The β-coefficients for the three non-*ortho*-substituted PCB congeners (PCB 81, PCB 126, and PCB 169) were not plotted with regard to a high proportion of samples with concentrations < LOD. The mono-*ortho*-substituted PCBs (congeners 105, 156, 167, 189, 157, 123, and 114; TEFs = 0.00003) are distributed along the line of best fit (*y* = 0.461*x* – 0.003; *R*^2^ = 0.797; *p* = 0.001). When we included β-coefficients for both PCB and TCDD (coordinates –1.101 for thyroid volume and –0.508 for FT_4_), we obtained the equation *y* = 0.459*x* – 0.003 (*R*^2^ = 0.999; *p* = 0.001). The slopes of these two equations were not statistically different, meaning that the lower end of the PCB best fit has a value similar to that of TCDD. This analysis suggests continuity between a dioxin-like and a non-dioxin-like effect. This conforms with the four orders of magnitude difference between TEFs for TCDD and most DL-PCBs.

## Discussion

Although some potential environmental hazards involve significant exposure to only a single compound, most instances of environmental contamination involve concurrent or sequential exposures to a mixture, which may induce similar or dissimilar effects over exposure periods ranging from short-term to lifelong (U.S. EPA 2000). Interest in the potential effect of chemical mixtures has increased significantly in the last decade (European Commission 2010; International Programme on Chemical Safety 2009; Kortenkamp et al. 2007). In this context, study tools such as the relative potency factor method have been developed. This approach uses empirically derived scaling factors based on toxicity studies of the effect in combination with exposure conditions of interest in the assessment (U.S. EPA 2000) and is the backbone of our study. The TEF method is a variation of the relative potency factor method (U.S. EPA 2000) and deals with the mixture toxicity of DLCs. The DLCs may serve as a prototype example of mixture toxicity (Van den Berg et al. 2006). Relevant studies with DLCs were reviewed extensively by Haws et al. (2006) within the framework of the TEF concept. One of the aims of the present study was to place our results in context of this vast body of scientific knowledge. As far as we know, the present study is the first human *in vivo* analysis of REPs of individual mixture components after exposure to DLCs.

Our study has several unique methodological aspects. First, for most congeners evaluated, we obtained six REP values, which were derived from the BMC, BMCL, and regression coefficient (β) approach for two end points, thyroid volume and serum FT_4_ concentration. The results of the three approaches are so closely interrelated that any of them can be used. A second aspect is that the potential effects of mixture components need to be accounted for. Multivariable regression analysis showed that the contribution of confounding congeners to the final outcome was negligible. Therefore, we did not adjust for the confounding congeners when calculating REPs (we adjusted only for age and sex). This approach is supported by differences in congener-specific mechanisms of action leading to their independent action.

With regard to our study design, several issues should be considered, the first of which is the selection of end points for exposure–effect analysis. We chose two thyroid biomarkers, serum FT_4_ concentration and thyroid volume, because thyroid pathology is the most prominent of the specific toxicological and biological noncancer health effects reported in DLC-exposed animals and humans (Boas et al. 2009; Crofton et al. 2005; Langer 2010; Rádiková et al. 2008; Zoeller 2007, 2010; Zoeller et al. 2002). Yang et al. (2010) suggested using a decrease in T_4_ as a prospective biomarker for generating a new human TEF scheme for DL-PCBs, noting that a decrease in circulating T_4_ is the only consistent biomarker for both DL- and NDL-PCBs. This is important because noncoplanar PCBs elicit a diverse spectrum of non-AhR–mediated toxic responses in humans and animals (Yang et al. 2010). In agreement with this, our results (Figure 2) demonstrate the association between the two thyroid end points using both DL-PCB and NDL-PCB data. Another issue to consider is dose additivity, which is assumed by the TEF method. In a short-term study using thyroid hormone–disrupting chemicals in rats, Crofton et al. (2005) observed both dose additivity and synergism depending on chemical dose. However, it is not known whether this would apply to our long-term, low-dose, human exposure scenario.

The second thyroid biomarker we evaluated in the present study is thyroid size. Estimation of thyroid volume is generally considered to be important in several pathologic situations, such as iodine deficiency goiter, thyroiditis, and multinodular goiter (Hegedüs 1990). In a study by Hansen et al. (2004), regression analysis suggested that serum thyroid-stimulating hormone, serum FT_4_, sex, age, smoking, and body mass index each played a small but significant role in variation of thyroid volume. We have been the only group that has extensively exploited this biomarker in studying effects of PCBs in humans (Langer 2010; Rádiková et al. 2008); thus, using thyroid volume in the present study was a logical continuation of our previous studies.

A significant finding in the present study is that the exposure to the index chemical, TCDD, and most DLCs was associated with a decrease in both thyroid volume and serum FT_4_ concentration. The FT_4_ shift is consistent with an observation in a community exposed to dioxin-like congeners (Bloom et al. 2006). In contrast, in our study, associations with PCB exposure varied slightly and were much smaller in magnitude. The parameter increases we observed for the most abundant NDL-PCBs agree with our previous results for FT_4_ and thyroid volume (Langer 2010; Rádiková et al. 2008) and for FT_4_ in anglers (Bloom et al. 2009).

A second important issue is the mode of action of the index chemical and of the congeners studied. In REP studies, similarity of the mode of action justifies the inclusion of a compound in the TEF concept for DLCs. The inclusion criteria include a structural relationship to TCDD, binding to the AhR, an AhR-mediated biological or toxic response, and persistence and accumulation in the food chain (Van den Berg et al. 2006). However, at present, there is no published evidence that long-term morphological changes of the thyroid gland and hormonal shifts—chosen as end points in this study—are exclusively AhR–mediated processes. We previously described a biphasic association between serum concentration of a mixture of PCBs and FT_4_ (i.e., negative association in the category of PCB levels < 530 ng/g vs. a positive association in the category of PCB levels of 531–25,000 ng/g) (Langer et al. 2007c); that association make even more difficult assigning a mode of action in humans exposed to complex environmental mixtures of DLCs and NDL-PCBs. In addition, there is no agreement on the presence of possible effects of DLCs on thyroid function at environmental exposure levels (Johnson et al. 2001; Pavuk et al. 2003).

In the present study, the REPs calculated via two different approaches—one based on thyroid morphology and the other on thyroid hormonal end point—showed consistent results. In spite of using a design different from those of published REP studies, as well as the unique scenario of our study, most of our REPs, especially those for dioxins and thyroid volume, fit well within the ranges of published REPs (Haws et al. 2006) (Table 1). In plots of log REPs for thyroid volume (Figure 1A) or FT_4_ (Figure 1B) versus log TEFs, however, the best fit is markedly shifted in the direction of our REPs. This is more pronounced for FT_4_, which may be interpreted as a greater sensitivity of this end point compared with thyroid volume or with end points leading to the assigned TEF values.

One strength of our study is that it is based on changes of two human thyroid parameters with apparently completely different pathogenesis, but whose results largely agree. Another strength is that we used actual serum concentrations of compounds that reliably reflect systemic body burden, rather than data on daily intake. A weakness of our study is that we worked with exposure to a mixture of chemicals with different potencies and likely different modes of action, compared with an exposure scenario under laboratory conditions that takes into account a single chemical. Further, single time exposure data does not necessarily reflect the whole exposure history of each participant. Another weakness of our study was that the prevalence of concentrations < LOD was high for some compounds, and this likely limited our statistical precision. In spite of the shortcomings of this study, the REPs we determined should be considered in updating the present TEFs with regard to long-term, low-dose exposure of humans instead of relatively short-term animal studies.

## Correction

In the manuscript originally published online, several errors resulted from the incorrect calculation of β coefficients. *a*) PCB 105 data for FT_4_ in Table 1 and Figure 1B were based on β coefficients calculated from concentrations given in nanograms per gram and compared with the β coefficient for TCDD, which was calculated from concentrations in picograms per gram. *b*) In Figure 2, the β coefficients for PCB 181, PCB 126, and PCB 169 were calculated from picograms per gram units instead of nanograms per gram units; the correct values were extremely low and have thus been omitted. These errors have been corrected here and do not affect the conclusions of the paper.

## Supplemental Material

(651 KB) PDF

## References

[r1] Akins JR, Waldrep KJT, Bernert JR (1989). The estimation of total serum lipids by a completely enzymatic ‘summation’ method.. Clin Chim Acta.

[r2] Bloom M, Vena J, Olson J, Moysich K. (2006). Chronic exposure to dioxin-like compounds and thyroid function among New York anglers.. Environ Toxicol Pharmacol.

[r3] Bloom MS, Vena JE, Olson JR, Kostyniak PJ (2009). Assessment of polychlorinated biphenyl congeners, thyroid stimulating hormone, and free thyroxine among New York State anglers.. Int J Hyg Environ Health.

[r4] Boas M, Main KM, Feldt-Rasmussen U (2009). Environmental chemicals and thyroid function: an update.. Curr Opin Endocrinol Diabetes Obes.

[r5] Brown DJ, Chu M, Van Overmeire I, Chu A, Clark GC (2001). Determination of REP values for the Calux® bioassay and comparison to the WHO TEF values.. Organohalogen Compounds.

[r6] Brunn J, Block U, Ruf G, Bos I, Kunze WP, Scriba PC (1981). Volumetrie der Schilddrüsenlappen mittels real-time Sonographie. Dtsch Med Wochenschr.

[r7] Crofton KM Craft ES Hedge JM Gennings C Simmons JE Carchman RA 2005 Thyroid-hormone-disrupting chemicals: evidence for dose-dependent additivity or synergism. Environ Health Perspect 113 1549 1554; 10.1289/ehp.8195 [Online 21 July 2005] 16263510 PMC1310917

[r8] Crump KS (1995). Calculation of benchmark doses from continuous data.. Risk Anal.

[r9] Dedik L (2012). CTDB_BMD (Clinical Trials Database– Benchmark Dose).. https://dl.dropbox.com/u/2951588/CTDB_BMD_inst.exe.

[r10] European Commission (2010). State of the Art Report on Mixture Toxicity: Final Report. Study Contract No. 070307/2007/485103/ETU/D.1. Brussels:Directorate General Environment, European Commission.. http://ec.europa.eu/environment/chemicals/pdf/report_Mixture%20toxicity.pdf.

[r11] Fattore E, Chu I, Sand S, Fanelli R, Falk-Filippson A, Håkansson H. (2004). Dose-response assessment using the benchmark dose approach of changes in hepatic EROD activity for individual polychlorinated biphenyl congeners.. Organohalogen Compounds.

[r12] Hansen PS, Brix TH, Bennedbaek FN, Bonnema SJ, Kyvik KO, Hegedüs L (2004). Genetic and environmental causes of individual differences in thyroid size: a study of healthy Danish twins.. J Clin Endocrinol Metab.

[r13] Haws LC, Su SH, Harris M, Devito MJ, Walker NJ, Farland WH (2006). Development of a refined database of mammalian relative potency estimates for DLCs.. Toxicol Sci.

[r14] Hegedüs L. (1990). Thyroid size determined by ultrasound. Influence of physiological factors and non-thyroidal disease.. Dan Med Bull.

[r15] International Programme on Chemical Safety 2009 Assessment of Combined Exposures to Multiple Chemicals: Report of a WHO/IPCS International Workshop. Harmonization Project Document 7 Geneva:World Health Organization, Available: http://www.who.int/ipcs/methods/harmonization/areas/workshopreportdocument7.pdf [accessed 16 April 2013].

[r16] Johnson E, Shorter C, Bestervelt L, Patterson D, Needham L, Piper W (2001). Serum hormone levels in humans with low serum concentrations of 2,3,7,8-TCDD.. Toxicol Ind Health.

[r17] Jursa S, Chovancová J, Petrík J, Lokša J. (2006). Dioxin-like and non-dioxin-like PCBs in human serum of Slovak population.. Chemosphere.

[r18] Koˇcan A, Drobna B, Petrik J, Jursa S, Chovancova J, Conka K (2004). Human exposure to PCBs and some other organochlorines in Eastern Slovakia as a consequence of former PCB production.. Organohalogen Compounds.

[r19] Kortenkamp A Faust M Scholze M Backhaus T. 2007 Low-level exposure to multiple chemicals: reason for human health concerns? Environ Health Perspect 115 suppl 1 106 114; 10.1289/ehp.9358 [Online 8 June 2007] 18174958 PMC2174412

[r20] Langer P. (2010). The impacts of organochlorines and other persistent pollutants on thyroid and metabolic health.. Front Neuroendocrinol.

[r21] Langer P, Koˇcan A, Tajtáková M, Koška J, Rádiková Z, Kšinantová L (2008). Increased thyroid volume, prevalence of thyroid antibodies and impaired fasting glucose in young adults from organochlorine cocktail polluted area: outcome of transgenerational transmission?. Chemosphere.

[r22] Langer P, Koˇcan A, Tajtakova M, Petrik J, Chovancova J, Drobna B (2005). Human thyroid in the population exposed to high environmental pollution by organochlorinated pollutants for several decades.. Endocr Regul.

[r23] Langer P, Koˇcan A, Tajtaková M, Petrík J, Chovancová J, Drobná B (2007a). Fish from industrially polluted freshwater as the main source of organochlorinated pollutants and increased frequency of thyroid disorders and dysglycemia.. Chemosphere.

[r24] Langer P, Koˇcan A, Tajtáková M, Rádiková Z, Petrík J, Koska J (2007b). Possible effects of persistent organochlorinated pollutants cocktail on thyroid hormone levels and pituitary–thyroid interrelations.. Chemosphere.

[r25] Langer P, Tajtáková M, Koˇcan A, Petrík J, Koška J, Kšinantová L (2007c). Thyroid ultrasound volume, structure and function after long-term high exposure of large population to polychlorinated biphenyls, pesticides and dioxin.. Chemosphere.

[r26] Langer P, Tajtaková M, Koˇcan A, Vlcek M, Petrik J, Chovancova J (2006). Multiple organochlorine pollution and the thyroid.. Endocr Reg.

[r27] Ozgen A, Erol C, Kaya A, Ozmen MN, Akata D, Akhan O (1999). Interobserver and intraobserver variations in sonographic measurement of thyroid volume in children.. Eur J Endocrinol.

[r28] Pavuk M, Schecter AJ, Akhtar FZ, Michalek JE (2003). Serum 2,3,7,8-tetrachlorodibenzo-*p*-dioxin (TCDD) levels and thyroid function in Air Force veterans of the Vietnam War.. Ann Epidemiol.

[r29] Petrik J, Drobna B, Pavuk M, Jursa S, Wimmerova S, Chovancova J. (2006). Serum PCBs and organochlorine pesticides in Slovakia: age, gender, and residence as determinants of organochlorine concentrations.. Chemosphere.

[r30] Rádiková Z, Tajtáková M, Koˇcan A, Trnovec T, Seböková E (2008). Possible effects of environmental nitrates and toxic organochlorines on human thyroid in highly polluted areas in Slovakia.. Thyroid.

[r31] Turner WE, DiPietro ES, Cash TP, McClure PC, Patterson DG, Shirkan H (1994). An improved SPE extraction and automated sample cleanup method for serum PCDDs, PCDFs, and coplanar PCBs.. Organohalogen Compounds.

[r32] Ukropec J, Radikova Z, Huckova M, Koska J, Koˇcan A, Sebokova E (2010). High prevalence of prediabetes and diabetes in a population exposed to high levels of an organochlorine cocktail.. Diabetologia.

[r33] U.S. EPA (U.S. Environmental Protection Agency) (1994). Method 1613: Tetra- through Octa-Chlorinated Dioxins and Furans by Isotope Dilution HRGC/HRMS (Revision B).. http://yosemite.epa.gov/water/owrccatalog.nsf/9da204a4b4406ef885256ae0007a79c7/d2967c045a3b082c85256b0600723f4d!OpenDocument.

[r34] U.S. EPA (U.S. Environmental Protection Agency) (1999). Method 1668, Revision A: Chlorinated Biphenyl Congeners in Water, Soil, Sediment, and Tissue by HRGC/HRMS.. http://www.caslab.com/EPA-Methods/PDF/1668a.pdf.

[r35] U.S. EPA (U.S. Environmental Protection Agency) (2000). Supplementary Guidance for Conducting Health Risk Assessment of Chemical Mixtures. EPA/630/R-00/002.. http://cfpub.epa.gov/ncea/cfm/recordisplay.cfm?deid=20533.

[r36] Van den Berg M, Birnbaum LS, Denison M, De Vito M, Farland W, Feeley M (2006). The 2005 World Health Organization reevaluation of human and mammalian toxic equivalency factors for dioxins and dioxin-like compounds.. Toxicol Sci.

[r37] Yang JM, Salmon AG, Marty MA (2010). Development of TEFs for PCB congeners by using an alternative biomarker—thyroid hormone levels.. Regul Toxicol Pharmacol.

[r38] Zoeller TR (2007). Environmental chemicals impacting the thyroid: targets and consequences.. Thyroid.

[r39] Zoeller TR (2010). Environmental chemicals targeting thyroid.. Hormones.

[r40] Zoeller TR, Dowling AL, Herzig CT, Iannacone EA, Gauger KJ, Bansal R (2002). Thyroid hormone, brain development, and the environment.. Environ Health Perspect.

