# Supplementary material for: Relative Effect Potency Estimates of Dioxin-like Activity for Dioxins, Furans, and Dioxin-like PCBs in Adults Based on Two Thyroid Outcomes
Source: Environ Health Perspect. 2013 May 10;121(8):886–92. doi: 10.1289/ehp.1205739 (PMC3734489; doi:10.1289/ehp.1205739)
Supplement: (651 KB) PDF [file ehp.1205739.s001.pdf]

Supplemental Material

Relative Effect Potency Estimates of Dioxin-like Activity for Dioxins, Furans, and Dioxin-like PCBs in Adults Based on Two Thyroid Outcomes

Tomáš Trnovec, Todd A. Jusko, Eva Šovčíková, Kinga Lancz, Jana Chovancová, Henrieta Patayová, Ľubica Palkovičová, Beata Drobná, Pavel Langer, Martin Van den Berg, Ladislav Dedik, and Soňa Wimmerová

Table of Contents

Supplemental Material, Table S1. The BMCs and BMCLs of TCDD calculated for thyroid volume as endpoint and simultaneous exposure to individual congeners ……………….page 2

Supplemental Material, Table S2. The median and mean serum concentrations, in pg WHO TEQ/g lipid of DLCs in the 320 study subjects. Non-ortho DL-PCBs are represented by a sum of congeners #77, 81, 126, and 169 ……………………………………………………..page 3

Supplemental Material, Table S3. Mean and median lipid adjusted serum concentration of PCDD, PCDF, DL- and NDL-PCB congeners, number of samples with concentration >LOD and number of samples with concentration >LOD overlapping with TCDD >LOD ……….page 4

Supplemental Material, Table S4. Results of multiple regression with backwards elimination with serum PCDD and PCDF congeners as independent and thyroid volume or FT4 as dependent variables……………………………………………………………………………page 6

Supplemental Material, Table S5. Regression coefficients β characterizing association between thyroid volume and FT4 outcomes and exposure to most abundant NDL-PCBs calculated from all concentration data >LOD. Gender and age of each subject were taken into account as confounders.…………………………………………………………………….....page 8

Supplemental Material, Table S1. The BMCs[a] and BMCLs[b] of TCDD calculated for thyroid volume as endpoint and simultaneous exposure to individual congeners[c]

		Confounders[d]			
BMCs	BMCLs	Confounder 3	Confounder 4	Confounder 5	Confounder 6
2.053	0.760				
2.061	0.785	123678-HxCDF			
2.061	0.785	123678-HxCDF	123478-HxCDF		
2.019	0.676	123478-HxCDF			
2.137	0.945	12378-PeCDD			
1.675	0.483	123678-HxCDD			
2.053	0.751	OCDD			
2.128	0.936	12378-PeCDD	123678-HxCDD		
1.751	0.634	12378-PeCDD	1234678-HpCDD		
1.751	0.634	12378-PeCDD	OCDD		
1.675	0.483	123678-HxCDD	1234678-HpCDD		
2.044	0.726	123678-HxCDD	OCDD		
1.667	0.458	1234678-HpCDD	OCDD		
2.128	0.936	12378-PeCDD	123678-HxCDD	1234678-HpCDD	
1.667	0.458	1234678-HpCDD	OCDD	123678-HxCDD	
1.734	0.609	12378-PeCDD	1234678-H[c]pCDD	OCDD	
2.128	0.928	OCDD	123678-HxCDD	12378-PeCDD	
2.120	0.911	12378-PeCDD	123678-HxCDD	1234678-HpCDD	OCDD

[a]Benchmark concentrations.

[b]Benchmark concentration lower confidence limits.

[c]The BMCs and BMCLs are expressed as pg/g fat.

[d]Various combinations of PCDD and PCDF congeners (confounders 3-6) were entered into the model besides the gender and age (confounder 1 and 2, respectively).

Supplemental Material, Table S2. The median and mean serum concentrations, in pg WHO TEQ/g lipid of DLCs in the 320 study subjects. Non-ortho DL-PCBs are represented by a sum of congeners #77, 81, 126, and 169

	Mean ± SD	Median
PCDDs	2.7 ± 2.8	1.5
PCDFs	7.1 ± 5.9	5.9
Non-orthoDL-PCBs	17.0 ± 24.8	10.5

Supplemental Material, Table S3. Mean and median lipid adjusted serum concentration of PCDD[a], PCDF[b], DL[c]- and NDL[d]-PCB congeners[e], number of samples with concentration >LOD [f] and number of samples with concentration >LOD overlapping with TCDD >LOD.

	Samples >LOD					Samples >LOD overlapping with TCDD >LOD				
	N	Mean±SD	Min	Median	Max	N	Mean±SD	Min	Median	Max
PCDDs										
2378-TCDD	70	1.731±1.091	0.562	1.506	6.439	70	1.731±1.091	0.562	1.506	6.439
12378-PeCDD	132	2.778±1.365	0.666	2.452	8.146	63	3.005±1.438	0.666	2.716	8.146
123478-HxCDD	80	2.714±1.411	0.769	2.400	7.799	42	2.764±1.448	1.244	2.466	7.799
123678-HxCDD	286	9.230±5.514	2.530	8.423	52.254	70	11.572±7.608	4.121	9.812	52.254
123789-HxCDD	75	3.381±1.920	0.676	3.068	9.097	31	3.241±1.671	1.037	3.068	8.411
1234678-HpCDD	316	16.183±11.565	0.592	13.003	75.356	70	19.869±11.721	2.371	17.504	55.453
OCDD	319	111.49±70.002	6.628	95.835	464.65	70	138.18±65.502	34.376	127.065	353.64
PCDFs										
2378-TCDF	43	1.937±1.922	0.421	1.406	11.326	21	1.378±1.197	0.421	1.258	5.728
12378-PeCDF	13	2.054±1.355	0.845	1.512	5.477	7	1.324±0.542	0.845	1.255	2.396
23478-PeCDF	318	19.237±17.627	3.068	15.470	166.69	70	23.731±14.183	9.041	18.951	85.62
123478-HxCDF	311	6.680±3.896	1.345	5.902	33.043	70	7.618±3.408	3.842	6.923	20.911
123678-HxCDF	312	6.235±2.613	1.373	5.859	17.956	69	6.997±2.538	2.835	6.455	15.544
234678-HxCDF	51	2.057±0.837	0.364	1.834	4.942	16	1.891±0.621	1.152	1.613	3.276
1234678-HpCDF	315	4.362±3.033	0.095	3.769	31.387	70	3.922±1.881	0.971	3.381	9.435
1234789-HpCDF	2	0.958±0.110	0.88		1.035					
OCDF	80	3.221±3151	0.308	2.652	25.865	21	2.279±1.695	0.308	2.04	8.619
DL-PCBs										
PCB 81	241	11.238±21.495	0.336	5.199	248.195	59	13.090±18.955	1.001	6.288	99.982
PCB 126	319	141.49±225.44	4.023	83.005	2525.74	70	225.47±248.81	21.035	143.87	1584.65
PCB 169	320	96.257±116.04	12.15	65.414	1411.29	70	1152.1±101.51	22.392	79.719	704.467
PCB 105	276	12.62±27.133	0.361	4.057	272.25	68	18.581±26.62	0.964	8.754	127.179
PCB 114	315	3.2±6.379	0.133	0.954	48.999	69	4.678±6.251	0.505	2.863	34.92
PCB 118	301	61.734±118.33	3.353	24.786	1097.59	69	94.029±120.001	6.88	50.074	598.7
PCB 123	276	1.337±3.518	0.0498	0.366	40.506	68	1.66±2.414	0.123	0.729	13.613
PCB 156	315	36.764±57.776	1.300	19.893	456.408	69	42.553±38.811	1.300	24.717	179.284
PCB 157	315	4.415±8.007	0.4015	2.215	70.631	69	4.887±4.618	1.021	3.113	24.789
PCB 167	315	16.744±27.223	1.417	7.889	265.821	69	21.892±20.455	3.299	13.264	90.129
PCB 189	315	8.68±15.984	0.527	4.231	139.939	69	9.331±8.339	1.361	6.483	32.745
NDL-PCBs										
PCB 28	130	23.71±30.615	2.891	13.579	238.099	42	20.73±23.341	2.891	13.006	127.905
PCB 52	38	15.615±14.854	4.403	10.874	76.507	8	8.702±3.131	4.403	8.845	14.19
PCB 101	45	18.2±35.269	3.44	11.405	243.378	14	11.88±8.351	3.44	10.677	33.523
PCB 138	320	380.3±665.46	46.24	206.268	7203.68	70	437.45±391.81	72.77	275.46	1923.71

	Samples >LOD					Samples >LOD overlapping with TCDD >LOD				
	N	Mean±SD	Min	Median	Max	N	Mean±SD	Min	Median	Max
PCB 153	320	609.87±1032.6	80.359	345.376	11194.7	70	686.44±605.23	120.34	437.42	3030.2
PCB 170	320	249.95±484.74	28.744	124.81	5051.35	70	246.55±202.13	48.605	163.53	875.38
PCB 180	317	599.29±1076.54	72.1	319.2	10995.3	70	587.59±472.98	103.4	404.3	2133.1

[a]PCDDs: polychlorinated dibenzo-p-dioxins

[b]PCDFs: polychlorinated dibenzofurans

[c]DL-PCBs: dioxin-like polychlorinated biphenyls

[d]NDL-PCBs: non dioxin-like polychlorinated biphenyls

[e]Concentration of PCDD, PCDF and non ortho DL-PCB congeners (81, 126, 169) is in pg/g lipids and of other DL-PCB and NDL-PCB congeners in ng/g lipids.

[f]LOD: limit of detection

Supplemental Material, Table S4. Results of multiple regression with backwards elimination[a] with serum PCDD and PCDF congeners as independent and thyroid volume or FT4 as dependent variables.

Outcome and Model	Unstandardized Coefficients		
	β	SE[b]	Significance
**Thyroid volume and Model A**			
Initial model (Constant)	13.14	2.632	0
2378-TCDD	-1	0.934	0.289
12378-PeCDD	-0.124	0.72	0.863
123678-HxCDD	-0.054	0.148	0.717
1234678-HpCDD	-0.108	0.122	0.381
OCDD	0.023	0.019	0.229
Final model (Constant)	13.684	1.549	0
2378-TCDD	-1.333	0.807	0.104
**FT4 and Model B**			
Initial model (Constant)	15.985	1.280	0
2378-TCDD	-0.247	0.455	0.589
12378-PeCDD	0.022	0.351	0.950
123678-HxCDD	-0.041	0.072	0.571
1234678-HpCDD	-0.057	0.060	0.339
OCDD	0.015	0.009	0.115
Final model (Constant)	15.418	0.878	0
1234678-HpCDD	-0.081	0.048	0.099
OCDD	0.016	0.009	0.068
**Thyroid volume and Model C**			
Initial model (Constant)	13.044	2.417	0
2378-TCDD	-1.001	0.700	0.158
23478-PeCDF	-0.016	0.116	0.890
123478-HxCDF	0.805	0.509	0.119
123678-HxCDF	-0.713	0.535	0.188
1234678-HpCDF	-0.207	0.524	0.695
Final model (Constant)	13.684	1.549	0
123478-HxCDF	11.669	2.085	0.010
123678-HxCDF	0.848	0.319	0.021
**FT4 and Model D**			
Initial model (Constant)	17.537	1.072	0
2378-TCDD	-0.573	0.352	0.109
23478-PeCDF	0.022	0.048	0.646
123478-HxCDF	-0.080	0.274	0.772
123678-HxCDF	0.017	0.251	0.946
1234678-HpCDF	-0.017	0.130	0.896
Final model (Constant)	17.433	0.657	0
2378-TCDD	-0.558	0.322	0.088

[a]Results of multiple regression with backwards elimination when 2,3,7,8-TCDD, 1,2,3,7,8-PeCDD, 1,2,3,6,7,8-HxCDD, 1,2,3,4,6,7,8-HpCDD and OCDD serum concentrations were entered as independent variables and thyroid volume (Model A) or FT4 serum concentration (Model B) as dependent variables (n=62) and when 2,3,7,8-TCDD, 2,3,4,7,8-PeCDF, 1,2,3,4,7,8-HxCDF, 1,2,3,6,7,8-HxCDF and 1,2,3,4,6,7,8-HpCDF serum concentrations were entered as independent variables and thyroid volume (Model C) or FT4 serum concentration (Model D) as dependent variables (N=68).

[b]Standard error

Supplemental Material, Table S5. Regression coefficients β characterizing association between thyroid volume and FT4 outcomes and exposure to most abundant NDL-PCBs calculated from all concentration data >LOD. Gender and age of each subject were taken into account as confounders.

	Thyroid volume	FT4
PCB-28	-0.00299	-0.00479
PCB-52	-0.0182	-0.017
PCB-101	0.0075	-0.0081
PCB-138	0.00062	0.00029
PCB-153	0.00041	0.0002
PCB-170	0.00079	0.00052
PCB-180	0.00038	0.00023
